# Family planning knowledge, attitudes and practices in refugee and migrant pregnant and post-partum women on the Thailand-Myanmar border – a mixed methods study

**DOI:** 10.1186/s12978-016-0212-2

**Published:** 2016-08-19

**Authors:** Patricia Salisbury, Layla Hall, Sibylla Kulkus, Moo Kho Paw, Nay Win Tun, Aung Myat Min, Kesinee Chotivanich, Somjet Srikanok, Pranee Ontuwong, Supachai Sirinonthachai, François Nosten, Shawn Somerset, Rose McGready

**Affiliations:** 1 Shoklo Malaria Research Unit, Mahidol-Oxford Tropical Medicine Research Unit, Faculty of Tropical Medicine, Mahidol University, Mae Sot, Thailand; 2 School of Allied Health, Faculty of Health Sciences, Australian Catholic University, Brisbane, Australia; 3 Mahidol-Oxford Tropical Medicine Research Unit, Department of Clinical Tropical Medicine, Faculty of Tropical Medicine, Mahidol University, Bangkok, Thailand; 4 The Planned Parenthood Association of Thailand, Bangkok, Thailand; 5 Centre for Tropical Medicine and Global Health, Nuffield Department of Clinical Medicine, University of Oxford, Oxford, UK

**Keywords:** Family planning, Long acting contraception, Female sterilization, Intrauterine device, Refugee, Migrant, Cross-sectional survey, In depth interview, Focus group discussion

## Abstract

**Background:**

Lack of data in marginalized populations on knowledge, attitudes and practices (KAP) hampers efforts to improve modern contraceptive practice. A mixed methods study to better understand family planning KAP amongst refugee and migrant women on the Thailand-Myanmar border was conducted as part of an ongoing effort to improve reproductive health, particularly maternal mortality, through Shoklo Malaria Research Unit (SMRU) antenatal and birthing services.

**Methods:**

Cross-sectional surveys and focus group discussions (FGDs) in currently pregnant women; and in-depth interviews (IDIs) in selected post-partum women with three children or more; were conducted. Quantitative data were described with medians and proportions and compared using standard statistical tests. Risk factors associated with high parity (>3) were identified using logistic regression analysis. Qualitative data were coded and grouped and discussed using identified themes.

**Results:**

In January-March 2015, 978 women participated in cross-sectional studies, 120 in FGD and 21 in IDI. Major positive findings were: > 90 % of women knew about contraceptives for birth spacing, >60 % of women in the FGD and IDI reported use of family planning (FP) in the past and nearly all women knew where they could obtain FP supplies. Major gaps identified included: low uptake of long acting contraception (LAC), lack of awareness of emergency contraception (>90 % of women), unreliable estimates of when child bearing years end, and misconceptions surrounding female sterilization. Three was identified as the ideal number of children in the cross-sectional survey but less than half of the women with this parity or higher in the IDI actually adopted LAC leaving them at risk for unintended pregnancy. Discussing basic female anatomy using a simple diagram was well received in FGD and IDIs. LAC uptake has increased particularly the IUD from 2013–2015.

**Conclusion:**

Definitive contextual issues were identified during this study and a significant range of action points have been implemented in FP services at SMRU as a result, particularly in regard to the IUD. The importance of the role and attitudes of husbands were acknowledged by women and studies to investigate male perspectives in future may enhance FP practice in this area.

**Electronic supplementary material:**

The online version of this article (doi:10.1186/s12978-016-0212-2) contains supplementary material, which is available to authorized users.

## Background

Family planning (FP) is an essential component of Sustainable Development Goal (SDG) 3 and addressed specifically in SDG 3.7: “By 2030, ensure universal access to sexual and reproductive health-care services, including for family planning, information and education, and the integration of reproductive health into national strategies and programs” [1]. Marginalized populations such as refugees and migrants, often unreached by “national strategies and programs”, face unique barriers to information and access that would allow them to plan and space the number of children they desire. Indeed family planning is key to preventing the social, economic and health consequences that result from unintentional pregnancies [2]. Contraceptive benefits arise especially among those at higher risk for maternal, perinatal and child mortality including pregnancies: at very young (<18 years) and old (>34 years) maternal ages; at high parities; with short inter-pregnancy intervals; and those that would have ended in unsafe abortion [3]. Family planning for high risk pregnancies is estimated to reduce the risk of maternal death by up to 58 % [4].

Maternal mortality on the Thailand-Myanmar border in rural dwelling refugee (79:100,000 live births) and migrant (252:100,000 live births) populations [5], and the displaced in eastern Myanmar (721:100,000 live births) [6], remains high. In Myanmar UNICEF estimates maternal mortality of 250:100,000 live births [7]. A parity of four or more amongst refugee and migrant women has been identified as a major risk factor for mortality [5]. As has induced abortion, a problem recognized in migrant Burmese women in urban centres such as Mae Sot [8] and more recently for rural refugees and migrants who travel to Mae Sot [9].

Thailand’s FP program, rated highly in comparative studies in South-East Asia (SEA), leaves few women with an unmet need for FP (estimated at 5.3 % (3 · 0–8 · 9) in 2010), whereas in Myanmar the estimate was 4 times higher at 20 · 0 % (12 · 4–29 · 5) in sentinel sites [10]. In 2013 the health information system for the Thailand-Myanmar border refugees suggested the contraceptive uptake in Maela (MLA), the largest camp, was lower (21 %) compared to the average of all 10 refugee camps (46 %). There is a paucity of information on the use of long-acting reversible contraceptives (LARC) in the Thailand-Myanmar border area despite the protracted nature of the camps, but a 2013 study involving in-depth interviews of 31 intrauterine-device (IUD) users, 21 of whom were from MLA Refugee camp, portrayed positive experiences [11]. An older report from 2005–07 evaluating surgical services (2005–07) in the same camp confirmed 477 sterilizations were provided for women with a median age of 33 years and a median parity of 5 which at the time was indicative of a significant unmet need for the procedure [12]. Current data on migrants from the area are not collated into any single data collection point with a range of community based organizations and non-government organizations providing services. Mae Tao Clinic based in Mae Sot, Thailand, reported 7,445 and 6,733 family planning consultations in 2013 and 2014. A survey presenting data from 2006 and 2008 from Eastern Myanmar in 2,442 participants in an area with an estimated 61,114 individuals (12,223 women of reproductive age) reported an increase in modern methods of family planning from 23.9 to 45.0 % (PRR = 1.88 [95 % CI 1.63-2.17]), and a reduced unmet need for contraception from 61.7 to 40.5 % [13]. These figures are positive but 40 % of women reported they did not want more children and uptake amongst modern contraceptive users of highly effective contraception at the end of the program was low: IUD use 2.8 % (30/1,070) and sterilization 1.6 % (17/1070).

Little is known about FP knowledge, attitude and practices (KAP) in refugee and migrant women or whether these marginalized groups are different. The relatively closed nature of the refugee camp and more mobile nature of migrants may affect needs, access and uptake. A poor understanding of marginalized populations prevents effective service provision due to a lack of local insight sensitive to the cultural, social and educational values of women in remote and rural areas [14, 15]. A 2010 survey among community health providers on reproductive health along the Thailand-Myanmar border reported a lack of knowledge about reproductive health and FP as one of the biggest health issues in the communities [16]. The aim of this study was to determine family planning KAP of rural refugee and migrant women on the Thailand-Myanmar, to identify gaps, and to improve engagement with reproductive health services, particularly in helping women align their contraceptive choice with their expressed need.

## Methods

### Study design

The study included a combination of methods: cross-sectional surveys (Additional file 1), focus group discussions (FGDs) (Additional file 2), in-depth interviews (IDIs) and a review of family planning uptake amongst the population.

### Setting

Shoklo Malaria Research Unit (SMRU), located in Tak Province of Thailand, has provided maternity services to the refugee population since 1986 and to the rural migrant population since 1998. The oldest SMRU clinic is located 60 km north of Mae Sot in MLA refugee camp. MLA is home to an estimated 45,000 people displaced from Myanmar due to poor security, ethnic violence, loss of livelihoods, lack of opportunity, and poverty in their home villages. In MLA the main health provider (at the time of this study) was Première Urgence–Aide Médicale International (PU-AMI) and for FP services Planned Parenthood Association of Thailand (PPAT). Since 2001, providing funding was available, all methods of family planning could be delivered by PPAT including short acting (depoprovera, oral contraceptive pill, male and female condoms) and LAC (IUD, implant, female and male sterilization). There is a high uptake (more than 90 %) by pregnant women in MLA camp to SMRU antenatal care (ANC) and birth facilities where there are approximately 1,200 deliveries per year.

SMRU commenced basic health services for Burmese and Karen speaking migrants (from Myanmar and Thailand), mainly agricultural workers, in 1998 (estimated population of 200,000). Financial, legal, logistical, and linguistic barriers complicate access to the Thailand healthcare system for this population. SMRU has two birth facilities for migrants with more than 1,500 deliveries per year. The first opened at Wang Pha (WPA, 30 km North of Mae Sot) in December 2007 and the second at Maw Ker Thai (MKT, 65 km South of Mae Sot) in April 2010.

ANC services at all three sites include routine investigation and treatment of malaria, anaemia, HIV, syphilis, screening for gestational diabetes mellitus, ultrasound examination (since 2001), vitamin supplements, vaccination, health information and advice, and care of medical or obstetric complications. The care provided in the SMRU birth facilities is described in more detail in a recent publication on quality of care [17]. Post-partum sterilization is offered at all three sites but is reliant on having a skilled practitioner available to provide it. For women referred into the Thailand Public Hospital system for caesarean section, sterilization can be offered at the same time.

### Participants and study size

**Fig. 1:**
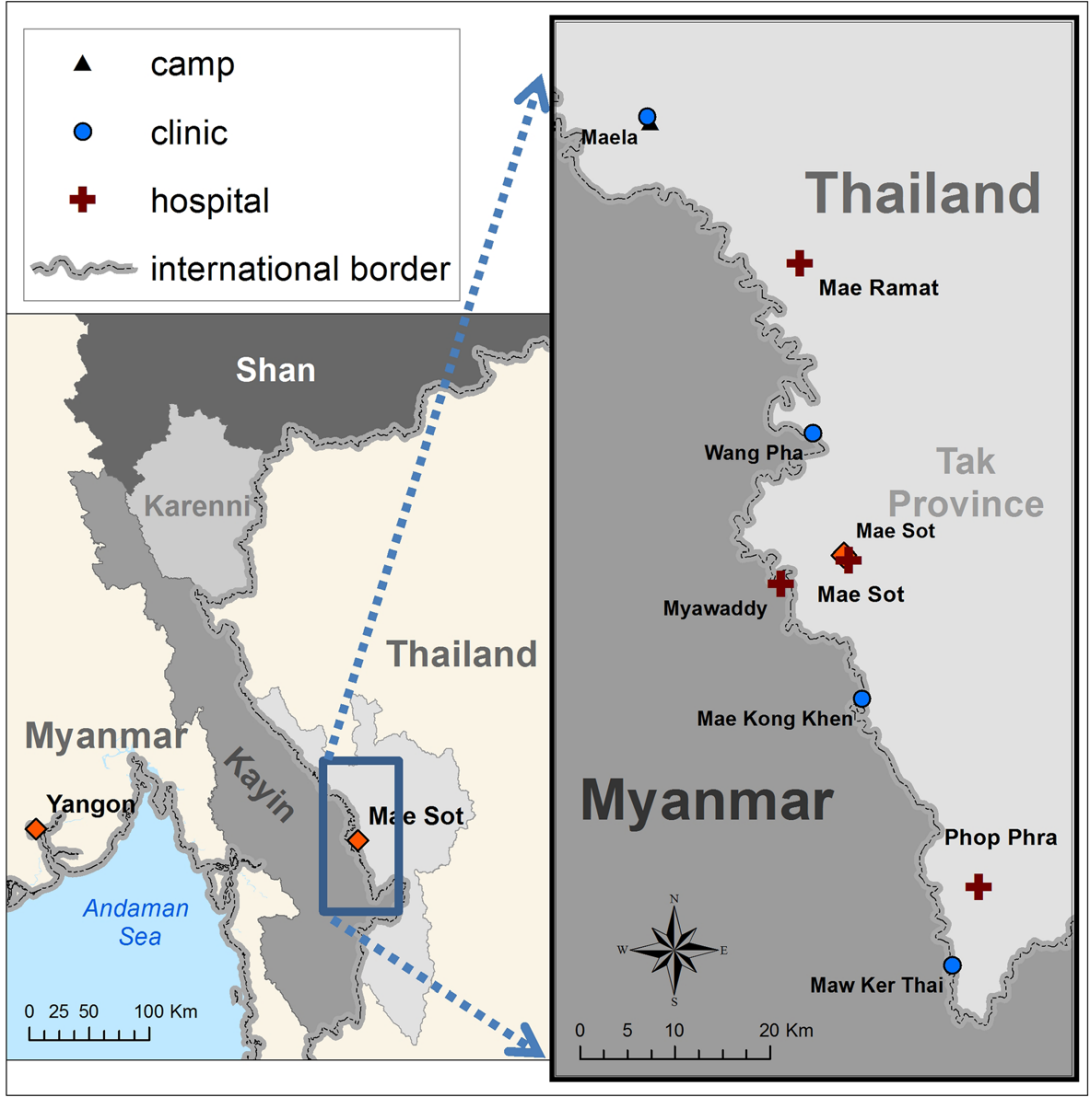
Location of the study sites. Credit to the Malaria Elimination Task Force Geo-unit

The study took place at the three SMRU ANCs (MLA, WPA and MKT) (Fig. 1) from Jan–March 2015. The women who attend these clinics come from a diversity of religious backgrounds. Due to limited ability to read fluently, as previously assessed in these clinics [18], a verbal explanation of the study was provided to all participants in their preferred language (Karen or Burmese) prior to each section of the study. Participants were free to leave the survey or discussions at any time. Those who agreed to take part were asked to sign or thumbprint a consent form.

### Cross-sectional surveys

A cross sectional survey comprising 10 questions (Additional file 1) was used to collect data on FP KAP. All women who were pregnant in January 2015 were eligible for inclusion. The December 2014 pregnancy registration data estimated a potential cohort of 1,052 and surveys were returned for 978 women (93 % of expected cohort).

Survey questions were verbally asked to each woman individually by ANC health workers trained in how to ask each question in the preferred language of each participant (Sgaw Karen, Poe Karen, or Burmese). Three questions (q5, q8, q9) presented scenarios that assessed attitudes in situations where reproductive health workers have observed difficulties when conducting post-partum FP counseling. The survey took approximately 10 minutes to complete.

### Focus group discussions (FGD)

FGDs were conducted with pregnant women at ANC at MLA, MKT and WPA. Group size was limited to five with an expected sample size of 120. Women could participate in both the cross-sectional survey and FGDs. Inclusion was based on meeting the specific criteria for each group including: language (Karen or Burmese), religion (Buddhist, Burman Muslim and Christian) and parity (nulliparous and multiparous with a parity of four or more i.e. those with a parity of one to three and likely to want more babies were not included). Facilitator and group factors were considered to create a setting in which the participants would feel comfortable discussing private or taboo topics, for example a young nulliparous woman might not feel comfortable speaking in a group with older, experienced mothers. Language groupings were used to reduce interruptions to the flow of the discussion. The facilitator, experienced in FGD techniques was fluent in Karen, Burmese and English.

The facilitator translated and interpreted for the researcher, who was the scribe. A series of questions (Additional file 2) guided discussion and maintained consistency across groups, however deviations were expected and encouraged based on spontaneous comments or questions. Qualitative and quantitative data were collected and no tape- or video-recordings were done. Transcripts were written immediately following the FGDs and reviewed by the facilitator for completeness and correctness.

FGDs were aimed at understanding the level of knowledge of pregnant women on the female reproductive system and LAC including female sterilization by tubal ligation (locally known as “steri”) and LARC with a focus on intra-uterine device (IUD). Implants were not the focus of the discussion due to their eight- fold higher cost compared to IUDs: Thai baht $1975 (US $65.00) for the 3-year single etonogestrol rod *versus* Thai baht $250 (US$8.00), compared in Dec 2014; raising sustainability issues but this did not prevent discussion of them during FGDs. A simple picture of the female reproductive system (screened for acceptability by the facilitator) and an expired Copper-T IUD were passed around the group and used to stimulate discussion. Participants were asked if they knew what the different parts of the female reproductive system were; if they knew what an IUD was and where an IUD would be placed, and which part of the reproductive system was operated on for sterilization. The dimensions of the picture were made to fit the IUD so women could try out different placements of the IUD onto the picture similar to a jigsaw puzzle.

A scenario that is repeatedly observed in practice was used to generate discussion on why high parity women at increased risk of maternal mortality fail to obtain FP [5]. This scenario involves a 35 year old highly parous woman (delivers her 6th alive child) who tells the midwife during postpartum FP counseling that she has completed her family and wants sterilization, but at two months post-partum she says she cannot come back to the clinic.

### In-depth interviews (IDI)

There were 21 post-partum women, purposively and consecutively asked to participate in an IDI. SMRU skilled birth attendants (SBA) and doctors routinely counsel women about FP options in the 24–72 h after delivery or pregnancy loss, before discharge. At the time of this counseling, selected women (parity of three of more) were invited to answer in more detail than usual through an IDI. All parties present at the IDI (SBA, clinic doctor, pregnant woman) were free to ask questions to help stimulate the discussion. Interview transcripts were recorded immediately and read back to all parties to confirm that the information was correct.

The IDIs elicited detailed information about the contraceptive choices each woman was planning to make and why. To give context to these conversations the outcomes of previous pregnancies including the number and sex of the children already born and still alive were recorded. Interviewers sought to understand the woman’s level of knowledge about the chosen method, and about other methods.

### Data sources

Data from the answers to the questionnaires were recorded onto Case Report Forms for each pregnant woman during the surveys before being transferred to an Excel database. Data from the cross-sectional surveys were coded before entry. Handwritten transcripts of the FGDs and IDIs were typed out in their entirety, in English into a Word (2007) document. Quantitative data from the FGDs were entered in an Excel spreadsheet. The contraceptives received by women in MLA Refugee Camp from PPAT and SMRU (IUD and sterilization only) were recorded and extracted from monthly reports. Limited SMRU data was available from migrant sites. Data from 2013 to 2015 were summarized.

### Data analysis

Quantitative data were expressed using median and range, and proportions with comparisons between sites (or between refugees and migrants), if applicable, tested using the Mann Whitney-U test or Chi-squared test using SPSS for Windows version 22.0 (SPSS, Inc., Chicago, Illinois, USA). Factors associated with a parity of three or more were evaluated by univariate analysis. Factors with a *p* < 0.10 in univariate analysis were entered into a forward logistic regression model with parity of three or more as the dependent variable, with associations presented as adjusted odds ratios (AOR) with their 95 % confidence intervals.

Data from FGDs were analysed using thematic analysis to identify and code themes emerging from the data [19, 20]. Two authors (LH and SK) independently analysed and coded data before comparing and discussing the themes identified. A third author (RM) was included to finalize the themes especially on comments that were difficult to code and when the two authors were unable to agree in which theme a specific comment should be placed.

Data on family planning uptake in MLA Refugee camp were reported as the number of new and continuing users per 1,000 live births because population denominators for the migrants are not known. PPAT clarifies the reporting: as funding has become difficult to find, having sufficient resources to confirm continuing users in MLA has also been difficult. The number of new users is more reliable.

## Results

### Cross-sectional surveys

Demographics differences between 414 MLA refugee and 564 migrant women in the cross-sectional surveys were observed (Table 1). Of note was the high infant/child mortality reported by more than 1 in 10 women in the migrant population. Cross-sectional survey responses (Table 2) suggest that the majority of women perceived three as the ideal number of children although over one-third of women thought that more than three children was ideal. Over 95 % of women proffered an age for menopause, but the range of suggested ages was large (20–70 years). Most (90 %) had never heard of emergency contraception, although most (93 %) knew that contraceptives can be used to space births and effective methods were required to prevent pregnancy. All contraceptive methods including LARC methods (implant, IUD) were mentioned but greater than 50 % of women nominated sterilization (51.2 %, 501/978).

**Table 1:** The baseline characteristics of refugee and migrant pregnant women in the cross-sectional survey

	Refugee camp	Total Migrants	*P* value	Migrant sites		*P* value
Site name	MLA *n* = 414	MKT & WPA *N* = 564	Refugee *vs* Migrant	MKT *N* = 292	WPA *N* = 272	MKT vs WPA
Age, years	25 [15–40]	25 [14–48]	0.618	25 [14–48]	25 [14–47]	0.982
Age ≥ 35 years, % (*n*)	10.2 (41/401)	14.8 (77/519)	0.046	18.4 (51/277)	10.7 (26/242)	0.018
Gravidity	2 [1–12]	2 [1–11]	0.076	2 [1–10]	2 [1–11]	0.821
Primigravidae, % (*n*)	27.9 (112/401)	34.9 (181/519)	0.027	36.5 (101/277)	33.1 % (80/242)	0.460
Primigravidae Age, years	20 [15–34]	20 [14–39]	0.755	20 [14–39]	20 [14–36]	0.337
Parity (if parity ≥1)	2 [1–10] *n* = 271	2 [1–8] *n* = 316	0.820	2 [1–8] *n* = 165	2 [1–8] *n* = 151	0.044^a^
Parity ≥4 (if parity ≥1), % (*n*)	18.1 (49/271)	18.0 (57/316)	1.000	21.2 (35/165)	14.6 (22/151)	0.144
History child death (if parity ≥1), % (*n*)	13.7 (37/271)	19.0 (60/316)	0.095	24.8 (41/165)	12.6 (19/151)	0.006
More than 1 marriage, % (*n*)	13.0 (52/401)	17.5 (91/510)	0.066	20.9 (58/277)	13.6 (33/242)	0.037
Literate (self-reported), % (*n*)	69.3 (278/401)	64.5 (335/519)	0.139	71.8 (199/277)	56.2 (136/242)	<0.001
Ethnic group, % (*n*)						
Sgaw or Poe Karen	83.3 (334)	53.0 (275)		44.8 (124)	62.4 (151)	
Burman	1.7 (7)	43.2 (224)		49.8 (138)	35.5 (86)	
Burman Muslim	12.7 (51)	0		0	0	
Other (e.g. Mon, PaOh, Rakhine, Kachin)	2.2 (9)	3.9 (20)	<0.001	5.4 (15)	2.1 (5)	<0.001
Husband age, years	28 [16–51]	28 [15–55]	0.542	28 [15–55]	28 [17–53]	0.793

Abbreviations: *MLA* Maela, *MKT* Maw Ker Thai, *WPA* Wang Pha, *vs* versus

Date are median [range] unless otherwise stated; ^a^MKT > WPA

**Table 2:** Knowledge, attitudes and practices related to family planning in refugee and migrant pregnant women: cross sectional survey results

	Refugee and Migrants			Migrant Sites Comparison		
Site name	MLA *n* = 414	Total migrant *N* = 564	*P* value	MKT *N* = 292	WPA *N* = 272	*P* value
Ideal number children	3 [1–10]	3 [1–9]	0.014	3 [1–9]	3 [1–7]	0.192
Think more than 3 children is ideal	42.8 (177/414)	35.1 (198/564)	0.017	34.6 (101/292)	35.7 (97/272)	0.792
Age menopause						
Did not answer % (*n*)	5.2 (22)	3.9 (22)	0.349	5.1 (15)	2.6 (7)	0.132
Median age (If answered)	35 [20–50]	40 [24–70]	<0.001	45 [24–70]	38 [25–60]	<0.001
How to space births						
Don’t know % (n)	1.4 (6)	2.7 (15)	0.030	4.1 (12)	1.1 (3)	0.034
Use contraceptive (if know) % (*n*)	93.9 (383/408)	99.5 (546/549)	<0.001	99.6 (279/280)	99.3 (267/269)	0.617
How to stop having births						
Don’t know % (*n*)	0.5 (2)	3.3 (18)	0.002	5.5 (16)	0.7 (2)	0.001
Effective method (if know)^a^ % (*n*)	98.3 (405/412)	99.5 543/546)	0.110	99.6 (275/276)	99.3 (268/270)	0.620
Use family planning before 1st baby						
Don’t know % (*n*)	6.3 (26/414)	5.7 (32/564)	0.684	8.2 (24/292)	2.9 (8/272)	0.010
No, you cannot (if know) % (*n*)	13.4 (52/388)	14.8 (79/532)	0.567	15.7 (42/268)	14.0 (37/264)	0.627
Type of family planning before 1st baby						
Yes, hormonal contraceptives +/− condoms^b^ % (*n*)	74.9 (251/335)	98.4 (440/447)	<0.001	97.7 (215/220)	99.1 (225/227)	0.278
Yes condoms only^b^ % (*n*)	25.1 (84/335)	1.6 (7/447)		2.3 (5/220)	0.9 (2/227)	
Termination if severe maternal heart disease						
Don’t know % (*n*)	8.0 (33/414)	8.3 (47/564)	0.906	8.2 (24/292)	8.5 (23/272)	1.000
Yes, (if know) % (*n*)	93.2 (355/381)	87.0 (450/517)	0.003	84.7 (227/268)	89.6 (223/249)	0.116
Termination if poor, parous, suddenly widowed						
Have baby and take care % (*n*)	86.9 (359)	66.8 (377)	<0.001	80.1 (234)	52.6 (143)	<0.001
Have baby and adopt % (*n*)	8.7 (36)	8.2 (46)		6.5 (19)	9.9 (27)	
Seek an abortion % (*n*)	4.4 (19)	25.0 (141)		13.4 (39)	37.5 (102)	
Heard of emergency contraception^c^						
Yes, (if know) % (*n*)	8.2 (24)	7.1 (40)^c^	0.542	7.8 (22)^c^	6.8 (18)	0.743

Abbreviations: *MLA* Maela refugee camp, *MKT* Maw Ker Thai migrant site, *WPA* Wang Pha migrant site

Data are median [min-max] unless otherwise stated; ^a^nominated an effective method (sterilization or long acting contraception); ^b^of women who replied ‘yes’ seven did not know what type of contraception could be used (one at MLA, six at MKT); ^c^two MKT women answered neither

The majority of women endorsed statements saying that a young (17 years), newly married woman could use FP before having her first baby, with most suggesting hormonal contraception; that a woman with heart disease could have a pregnancy termination to save her life; and that a recently widowed pregnant woman should continue the pregnancy and care for her child, though a minority chose adoption or induced abortion as preferred options. The suggestion to have an abortion was significantly higher at WPA (*p* < 0.001) the site closest to the urban centre of Mae Sot. Amongst women who suggested this, they explained it would be obtained from a traditional birth attendant 59.4 %, (95/160), by taking herbal medicine 16.3 % (26/160), or by going to a doctor 24.4 % (39/160). Very few women did not know where to obtain contraceptives (*n* = 18) and the three most commonly named sources of contraceptives were summarized for each site (Table 3).

**Table 3:** Three most common places name at each site of where women reported they could obtain contraceptives

MLA Refugee camp	MKT Migrant site	WPA Migrant site
PPAT clinics 66.5 % (290/436)	The market 33.5 % (155/462)	Health clinic in Thailand or Myanmar 35.1 % (195/555)
SMRU 25.5 % (111/436)	SMRU 31.0 % (143/462)	The market 27.2 % (151/555)
The market 4.6 % (20/436)	Thailand Hospital 20.1 % (93/462)	Thailand hospital 16.0 % (89/555)

*MLA* Maela, *MKT* Maw Ker Thai, *WPA* Wang Pha

There was no significant association between having a high parity (*P* > 3) and knowing that sterilization can be used to end fertility (Table 4).

**Table 4:** Factors associated with a parity >3 in refugee and migrant women on the Thailand Myanmar border

Variable examined	*N*	Parity >3	OR (95 % CI)	AOR (95 % CI)
		% (*n*)		
No history child death	823	6.2 (51)	Reference group	Reference group
History child death	97	56.7 (55)	2.166 (1.724-2.722), *p* < 0.001	17.539 (9.749-31.553), *P* < 0.001
Refugee	401	12.2 (49)	Reference group	Not included
Migrant	519	11.0 (57.0)	0.986 (0.940-1.034), *p* = 0.603	
Age <35	802	5.5 (44)	Reference group	Reference group
Age ≥35y	118	52.5 (56)	1.992 (1.646-2.410), *p* < 0.001	16.232 (9.355-28.165), *p* < 0.001
One marriage	777	9.7 (75)	Reference group	Reference group
More than one marriage	143	21.7 (31)	1.154 (1.055-1.261), *p* < 0.001	Not significant
Literate	613	9.0 (55)	Reference group	Reference group
Illiterate	307	16.6 (51)	1.092 (1.032-1.154), *p* = 0.001	Not significant
Ethnic Karen/Burman/Other^a^	869	10.9 (95)	Reference group	Reference group
Ethnic group Burman Muslim	51	21.6 (11)	1.291 (1.112-4.514) *p* = 0.024	2.699 (1.105-6.592), *p* = 0.029
Know sterilization can end fertility	901	11.4 (103)	Reference group	Not included
Don’t know	19	15.8 (3)	1.052 (0.864-1.280), *p* = 0.473	

^a^Comprising of Karen 10.8 % (66/609), Burman 12.6 % (29/231) and Other (0 of 29) pooled together

### Focus group discussions (FGDs)

A total of 120 women participated in the 24 FGDs, 11 at MLA, eight at MKT and five at WPA. Homogeneous focus groups were assembled around common religion, language, and parity as described earlier and eleven of the groups were reserved for primigravidae. Most women acknowledged contraceptive use in the past, predominantly the OCP or depo with only two women reporting IUD use. None reported using condoms, norplant or implanon. The activity with the diagram of the woman’s reproductive system and working out where the IUD should be placed was particularly well received. Most women reported that they had never seen this type of diagram before or that is was completely unknown to them and less than half knew which part of the reproductive system was affected in female sterilization.

#### Female sterilization: family and health

Two main themes emerged from 107 comments proffered as reasons for why a woman would undergo sterilization, namely family and health (selected responses Table 5). Family themed comments were mostly related to enough (or too many) children, or meeting the physical and financial demands of a family. Health comments were related to maternal age as a reason to have sterilization, but also involved misconceptions. Nearly half of the participants reported that sterilization would not cause problems for the woman. Of those who did expect problems from sterilization, many were concerned their ability to work and carry heavy things afterwards would be limited significantly, and others raised concerns related to religious or cultural beliefs. Domestic violence as a reason to be sterilized was nominated by three women.

**Table 5:** Selected responses from FGD related to female sterilization, use of IUD and remarriage

Category	Type of response	Responses
Female sterilization	Reasons for choosing female sterilization	“…*because if we have too many children we are not free to work, as we need to care for the baby.*” [multip, Buddhist, MLA]
		*“Maybe they have a difficult delivery like caesarean section so they get steri.”* [primip, Buddhist, WPA]
		*“She is very old and worries about going through pregnancy again.”* [primip, Buddhist, MKT]
	Misconceptions	“*My mother had a steri because she had an abnormal placenta.”* [primip, Buddhist, MKT] (After discussion the facilitator ascertained that the surgical procedure was a hysterectomy not a sterilization).
		*“I heard someone say you cannot walk and work for 3 years (after a steri).*” [primip, Buddhist, MKT]
	Beliefs / religious	“Allah *doesn’t like sterilization. If they get steri they don’t get to go to heaven, they go to sin.*” [Multip, Burman Muslim, MLA]
		From a woman who has four boys: “*After this pregnancy I will get a steri, but if I have a girl I need another child because I hear steri is bad…people in my village believe this.* (Can you explain?) *We are Buddhist and if we have four boys it is bad. Four boys will carry the coffin, which is not good. Four boys means one parent could die at any time so we need another son*.” [Multip, Buddhist, MKT]
IUD	Reasons for choosing IUD	“*Maybe some women live far and they cannot get to the clinic to buy/get medicine. This* (IUD) *is for 5 years so they don’t need to worry for 5 years. It’s easy for her*.” [Multip, Buddhist, MKT]
		“*Some have side effects of depo or pill so they can use this.*” [Primip, Buddhist, WPA]
	Beliefs/religious	“*Yes we can use this as we don’t kill the baby so Allah says it’s OK.*” [Multip, Burman Muslim, MLA]
	Positive reaction after seeing/touching IUD	“*Before I thought you could get problems like pain in the uterus. Because we have never seen the IUD we worry and we don’t trust, but now we know we can trust.”* [Multip, Burman Muslim, MLA]
Remarriage	Reasons to have a child after remarrying	*“I saw one woman who was old and didn’t want to get pregnant but her second husband* (usual terminology to express first remarriage) *said he wanted a child, so she did.” [Multip, Buddhist, MKT]*

Abbreviations: *depo* depoprovera, *IUD* intrauterine device, *Multip* multiparous, *Primip* primigravidae, *MLA* Maela Refugee camp, *MKT* Maw Ker Thai Migrants, *steri* sterilization, and *WPA* Wang Pha Migrants

The reasons nominated for a grand multiparous woman not returning for sterilization post-partum fell into four main categories: fearful, no childcare, husband not in agreement and access difficulties.

#### The intrauterine device (IUD): health and beliefs

Less than half of the women could correctly position the IUD in the diagrammatic representation of the female reproductive system. The themes of health and beliefs again emerged in relation to IUD use (selected responses Table 5). Reasons provided for women choosing the IUD as contraception included: longevity of effect, ease of use, and less side effects compared to other contraceptives. While most women did not think there would be problems with an IUD those who had health concerns mentioned pain or infection. Religious concerns were raised less frequently for the IUD compared to sterilization. Allowing women to see and touch the IUD during the FGD was associated with the expression of positive attitudes towards the IUD during the discussion.

#### Child bearing after remarriage: beliefs

Most women agreed that a woman would need to have a child after remarrying, with most of those citing the husband’s wants or needs as the main reason behind this (selected responses Table 5).

### In-depth Interviews (IDIs)

The two main themes that emerged from the IDIs were:

i) Lack of knowledge and fear/misconceptions:

*“I want a steri but I don’t know what it is.*” [gravida 4, para 4, migrant, MKT, post-partum haemorrhage at delivery]

*“I want to use IUD but I am afraid*”. [gravida 8, para 7, migrant, MKT, severe anaemia from post-partum haemorrhage at delivery]

*“…because I am old I will not get pregnant”.* [37 yo, gravida 6, para 6, refugee, MLA, ECV for breech]

*“I forgot OCP. I want to wait before the next baby. I am afraid of IUD because someone told me it can move and cause bleeding.”* [gravida 4, para 4, migrant, WPA, hepatitis B positive]

*“I am very afraid that after a steri I cannot lift heavy things. I carry 25 kg of rice two to three times a month, also charcoal”.* [gravida 6, para 6, refugee, MLA, gestational diabetes, malaria]

*“My sister had the operation and my sister and my husband told me not to get it because I will be unwell like her”*. [gravida 5, para 4, refugee, MLA, pre-eclampsia and preterm labour at 33 weeks gestation] On confirmation the operation was a caesarean section not a simple sterilization.

ii) Role of the Husband:

*“I was using depo but stopped because my new husband wants a child.”* [gravida 6 para 5, MKT, migrant, treated for soil transmitted helminth infection during pregnancy]

*“My husband does not agree for steri but after counseling we agree for IUD”.* [gravida 6, para 5, MLA, refugee, became pregnant because OCP ran out but does not want more children]

*“I want to use depo but my husband wants steri”* and after counseling, *“we agree for steri”*. [gravida 6, para 6, WPA, migrant, pregnant because forgot to come back for depo]

Complementary to the themes were the problems regarding past FP use and final FP choice in these selected cases. The 21 IDI participants had a high parity (median of 6; range 3–9). Over half 52.4 % (11) had children who had died (10 had one child die and one woman had three die). Most planned to have 5 (range 3–9) children, less than their own mothers (7; range 3–12) and less than half (42.9 %; 9/21), indicated the current pregnancy was planned. Most women (76.2 %; 16/21) had medical and/or obstetric problems, had used FP previously (76.2 %; 16/21) including two who used norplant but none who had used an IUD. Of the 16 who reported using contraceptives 50.0 % (8) reported side effects, 12.5 % (2) had forgotten to take the OCP and 25.0 % (4) reported having clinic access issues. There were 16 mothers who said they had completed their family, four were specific about needing either a girl or a boy, and one remained uncertain. Eleven women opted for LAC as their first FP choice and of the remaining 10, nine agreed to LAC after further counseling. However at one month post-partum 28.6 % (6) of women could not be traced, LAC uptake was confirmed in less than half (42.9 %; 9/21) of the women (five followed through with their first and four their second choice) and the remaining 6 women opted for Depo.

### Family planning statistics 2013 to 2015

To provide perspective to the mixed methods research, trends in family planning uptake for the population were collated and summarized from the two organizations involved with this service in MLA Refugee Camp (PPAT and SMRU for sterilization and IUD) (Additional file 3: Table S1). Comparable data collection for migrants and refugees were compared from 2013 to 2015: in MLA and MKT, LAC has increased mostly due to a rise in IUD uptake in the last one year (Table 6) but WPA has not seen a significant increase in IUD uptake.

**Table 6:** New long acting contraceptive uptake per 1,000 livebirths at each site for 2013–2015

	MLA Refugee camp			WPA Migrants			MKT Migrants		
Year	2013	2014	2015	2013	2014	2015	2013	2014	2015
live births, *n*	1171	1101	1076	705	751	842	666	670	688
IUD, per 1,000 live births	60	78	248		23	26			80
	(46–73)	(62–94)	(222–274)	0	(12–33)	(15–37)	0	0	(60–100)
Sterilization, per 1,000 live births	81	55	67		76	59		73	44
	(65–97)	(42–69)	(84–100)	0	(57–95)	(43–75)	0	(53–93)	(28–59)
Implant, per 1,000 live births	32	25	6						
	(22–42)	(15–34)	(1–10)	0	0	0	0	0	0
LAC Total, per 1,000 live births	173	158	337		99	86		73	124
	(152–195)	(136–180)	(309–366)	0	(77–120)	(67–104)	0	(53–93)	(99–148)

n.a. Records were destroyed with flooding

## Discussion

Context plays a major role in barriers and enablers of successful strategies to fill unmet family planning needs [21]. Worldwide over 200 million couples state a desire to delay pregnancy or cease fertility but have an unmet need for contraception, more so if they are amongst the poor, less educated, and rural residents of our globe, and this was reflected in the data collected during this study [22]. Needs for appropriate family planning in conflict affected areas are significant and recent research suggests use of LARCs are highly feasible [23]. Strengths and gaps in KAP towards FP in rural refugee and migrant women were identified in this study and provide an avenue for change, potentially impacting maternal mortality and morbidity in these marginalized populations [24, 25].

Pregnant women from all ethnic and religious groups were very open to FP discussion. Encouragingly, more than 90 % of women knew about contraceptives for birth spacing, and more than 50 % knew sterilization could end child bearing and believed it was acceptable for nulliparous women to use contraceptives, women knew where they could obtain FP supplies and more than 60 % of women in FGDs and IDIs reported use of FP in the past. This study does not reflect the views of single and unmarried women, and obtaining more information in this area could be very helpful to inform FP services [26]. This is especially important since a survey conducted with 15–24 year olds a decade earlier (2005–6) in Mae Ra Ma Luang and Mae La Oon refugee camps, approximately 300 km north of MLA, reported young people had very limited knowledge of reproductive health issues, with only about one in five correctly answering at least one question on reproductive health [27]. Sex education is not taught in schools and pregnant girls have previously been asked to stop school as they are thought to be a bad influence [28]. A more recent survey in 2011 in Mandalay City in Myanmar suggests access too and utilization of reproductive health services for youth is alarmingly poor.

Contraceptive choices and reported prior use were predominantly short acting hormonal contraceptives and no significant association was observed between having a high parity (*P* > 3) and knowing that sterilization can be used to end fertility. Fear was identified as a major barrier to sterilization in FGDs and IDIs. Emergency contraception was essentially unknown and there were misconceptions about the consequences and types of gynecologic operations (cesarean section and hysterectomy versus sterilization). Fear that an operation would prevent work afterwards was a serious concern because in rural areas if you cannot work, you cannot support your family. The fear is understandable in an environment that has only seen operations done for emergent indications: these patients usually are very sick and if they recover it is a long process. Estimates of when child bearing years are complete were generally unreliable. In the scenario of the suddenly widowed woman it was suggested that abortion was an option. Legal termination is only available for rape and life-saving medical indications in Thailand and Myanmar, but the answer suggests women will put themselves at risk to solve problems of unwanted pregnancy [29] and indeed this has been observed in clinical practice in this setting [9]. Consultations and negotiations with key members of the generally conservative local community [30] have commenced as a result of this study and as previously recommended from an evaluation of the availability, service delivery, and barriers to access to emergency contraceptive pills (ECPs) along the Thailand–Burma border [31].

While three children was the median ideal number of children in the cross-sectional survey, less than half of the participants in the IDI, who were all women with this parity or higher, ultimately adopted LAC. This occurred even though the women had a high rate of medical and obstetric problems, and these problems were brought into the post-partum family planning discussion. This low rate of LAC uptake is problematic and will likely lead to unintended pregnancies in the future for some of these high risk women. This finding diverges from recent observations in a developed country setting where personal history of pregnancy complications appeared to positively affect contraceptive choices [32]. The high loss to follow up at one month post-partum in the IDI women underscores the need for future programs to emphasize achieving effective FP before discharge. The FGDs also suggest that discussing basic anatomy is a beneficial tool in FP counseling in this setting, providing possibly unknown information and allaying fears about contraceptive methods [33]. The IDIs indicate that when the first choice of FP does not match the desired family size, further counseling about that choice may lead to a more reliable contraceptive method in this population. When local health workers gain knowledge and confidence in FP counseling they can motivate change as demonstrated by the increase in IUD uptake since the survey took place. This knowledge and confidence may explain in part the difference in LAC uptake between the refugee and migrants but supply of contraceptives by community based organizations may also be important however such data was not available.

Changes in service provision have been implemented in response to the findings of this survey (Table 7). For refugee and migrant women the cost of family planning is an important issue to address. In this case, the cost of family planning supplies but not health workers who can implement the work, has been met for a short time by a charitable donor but this is precarious in terms of sustainability [34]. The problem of displacement is increasing globally, with more refugees and displaced people than at any other time since the Second World War. Tens of thousands of Karen and Burmese from these camps have been legally resettled by the United Nations Refugee program in one of the largest resettlement programs to date, with the USA accepting more than 70,000 into their program [35]. Other countries that have also accepted thousands of this particular group of refugees include Australia, UK, The Netherlands, Nordic countries, and Canada, and the difficulties associated with health literacy and the fears and misconceptions in regard to family planning have travelled with them. Family planning programs that include resettled populations may have to modify practice compared to their population of native citizens [36, 37].

**Table 7:** Changes in service provision in regards to family planning following survey

Target	Change in service provision
Midwife training points	Benefits of LAC
	Health consequences of choosing methods with a higher risk of failure Risks of pregnancy in women over 35 years of age
	Sensitive counseling in women with a history of a child death
	Identify and address fears surrounding sterilization
	Clarify the word operation: take time to distinguish the small 1–2 cm wound of a female sterilization from others such as cesarean section
	Explanation of recovery time after a sterilization
	Use of the diagram with every one-on-one counseling
	Provision of contraception before discharge
	Benefit and importance of counseling women about tubal resection before cesarean section
Antenatal care counseling for women	FP counseling increased: more frequent and earlier messaging with encouragement to discuss the family size with the husband
	At 22 weeks all *P* ≥ 1 discuss the option of sterilization in the event of unexpected cesarean section
	At 22 weeks all *P* ≥ 1 discuss the option of sterilization/IUD and implant
Postnatal care counseling for women	If FP cannot be provided before discharge then follow-up and engagement at the first post-partum visit is required
Inclusion of men	Women are encouraged to bring their husbands to come to the clinic and discuss the family size, medical and obstetric problems
	Couple counseling particularly encouraged in parity >3 with the highest chance to provide this in the post-partum period

Abbreviations: *LAC* long acting contraception

There are several limitations to this study. Data were self-reported and women may not have remembered what contraception they took in the past. Some of the questions posed were of an ethical nature and women had the option to choose “don’t know” rather than express their opinion. There is an inherent risk of bias in any study and this we aimed to minimize by exploring KAP via different methodologies (cross-sectional survey, FGD and IDI). FGD can occasionally be dominated by one person but the high number of FGDs achieved saturation. The IDIs cannot be generalized to younger women with lower parity. A notable deficit was engagement of men who should be included in discussions of family culture since their opinion significantly impacts on their families. This theme emerged from the IDIs, where husbands were noted to both positively and negatively impact LAC uptake [38]. Male peer educators could be considered in this setting since disagreement by the husband was noted in one-third of the FGD comments as to why a high-risk woman may not have had a sterilization, and because in remarriage a woman may have more children than they desire [39].

## Conclusions

The present paper primarily represents the voices of mothers, who are indeed the main consumers of obstetric services. Many of the reflections noted in this study acknowledge the importance of the roles that fathers, families, community and culture play in decisions about family planning. The development of sustainable interventions to improve engagement with and uptake of family planning services is contingent on better understanding of barriers and facilitators across the full range of stakeholders locally. In particular, the perceived attitudes of husbands was reported as an important influence on decision making, and studies to investigate their perspectives directly may enhance the development of interventions to enhance family planning practices in this population.

## Additional files

Additional file 1: Cross sectional survey questions. (PDF 209 kb) Additional file 2: Focus group discussion. (PDF 192 kb) Additional file 3: Table S1. Contraceptive uptake in Maela Camp 2013 to 2015. (PDF 214 kb)

## Abbreviations

ANC: Antenatal clinic
Depo: Depo-medroxyprogesterone acetate
FGD: Focus group discussion
FP: Family planning
IDI: In-depth interview
IUD: Intra-uterine device
KAP: Knowledge, attitude and practice
LAC: Long acting contraception
LARC: Long acting reversible contraception
MKT: Mawker Tai migrant clinic
MLA: Maela refugee camp
OCP: Oral contraceptive pill
PPAT: Planned Parenthood Association of Thailand
SRH: Sexual and reproductive health
WPA: Wang Pha migrant clinic
