# Supplementary material for: Family planning knowledge, attitudes and practices in refugee and migrant pregnant and post-partum women on the Thailand-Myanmar border – a mixed methods study
Source: Reprod Health. 2016 Aug 19;13:94. doi: 10.1186/s12978-016-0212-2 (PMC4992227; doi:10.1186/s12978-016-0212-2)
Supplement: Additional file 1: — Cross sectional survey questions. (PDF 209 kb) [file 12978_2016_212_MOESM1_ESM.pdf]

number_______________

Cross sectional survey questions

1. What is the ideal number of children to have? |__|__|

2. At what age does a woman stop being able to get pregnant? |__|__|years or don't know|__|

3. What are the ways you can space your births? (cross any that apply)

Stop having sex [___][1] Use contraception (something to stop you from getting pregnant) [___][2]

Get a sterilization [___][3] Don't know [___][4] Other[___][5] _____________________________________

4. What are the ways to stop having children when you finished your family? (cross any that apply)

Stop having sex[___][1] Getting old[___][2] Getting a sterilization[___][3] Using contraception forever[___][4]

Don't know[___][5] Other[___][6]____

5. If Naw Paw and Saw Tha get married at 17 yo, can they have family planning before having their first baby?

YES[___][1] NO[___][2] Don't know[___][3] If yes go to 7, if No or don't know go to 8.

6. How would Naw Paw and Saw Tha do that?

Not having sex[___][1] Using OCP[2] Using Norplant or IUD[3] Other[4]______________________________________

7. Where can you go to get family planning?

In the market [___][1] PPAT[___][2] SMRU[___][3] PU-AMI[___][4] Other[5]__________________

8. If Tha Paw gets pregnant and has a heart problem that the pregnancy will make worse and can kill her, does she still need to have the baby? YES[___][1] NO[___][2] Don't know[___][3]

9. What are the choices if a friend gets pregnant (2 months) and thinks she already has too many children and her husband died suddenly in a motor bike accident?

Have the baby anyway and take care of it all by herself[___][1]

Have the baby and give it away[___][2] See the TBA for an abortion[___][3] See the Doctor for abortion[___][4]

Take a herbal treatment for abortion[___] [5](write here)____

10. Have you heard of emergency contraception/morning after pill (Show)? YES[___][1] NO[___][2] Don't know[___][3]
