# Supplementary material for: Family planning knowledge, attitudes and practices in refugee and migrant pregnant and post-partum women on the Thailand-Myanmar border – a mixed methods study
Source: Reprod Health. 2016 Aug 19;13:94. doi: 10.1186/s12978-016-0212-2 (PMC4992227; doi:10.1186/s12978-016-0212-2)
Supplement: Additional file 2: — Focus group discussion. (PDF 192 kb) [file 12978_2016_212_MOESM2_ESM.pdf]

Supplementary file 2

FGDs were conducted with pregnant women at ANC at MLA, MKT and WPA. Group size was limited to five with an expected sample size of 120. Women could participate in both the cross-sectional survey and FGDs. Inclusion was based on meeting the specific criteria for each group including: language (Karen or Burmese), religion (Buddhist, Burman Muslim and Christian) and parity (nulliparous and multiparous with a parity of four or more i.e. those with a parity of one to three and likely to want more babies were not included). The facilitator, an experienced researcher, fluent in Karen, Burmese and English, has a close affinity to the camp population and was married to a migrant worker. Qualitative and quantitative data was collected and no tape- or video-recordings were done. Transcripts were written immediately following the FGDs and reviewed by the facilitator for completeness and correctness.

Facilitator

The facilitator, was formerly employed for over 10 years as a skilled birth attendant at SMRU familiar with conducting these types of discussions, was fluent in Karen, Burmese and English, has a close affinity to the camp population and was married to a migrant worker.

Setting

Facilitator and group factors were considered to create a setting in which the participants would feel comfortable discussing private or taboo topics. Divisions based on parity and religion helped increase the comfort of the participants: a young nulliparous woman might not feel comfortable speaking in a group with older, experienced mothers. Language groupings were used to reduce interruptions to the flow of the discussion.

Method

After inclusion by language and religious groups, parity groups were also identified. We were interested in the extremes and chose to concentrate on nulliparous and multiparous (parity of four or more). This means that we excluded those with a parity of one to three because we most of these women would like more babies.

FGD involved the women, the facilitator (MKP) who translated and interpreted to the researcher who was the scribe. After a scripted welcome, introduction and thank you by the facilitator women were reminded that they were able to leave at any time and without consequence and encouraged to feel free to participate at any time. A series of questions guided discussion and maintained consistency across groups however deviations were expected and encouraged based on comments or questions. Qualitative and quantitative data was collected and no tape- or

video-recordings were done. Transcripts were written immediately following the FGDs and reviewed by the facilitator for completeness and correctness.

FGDs were aimed at understanding the level of knowledge of pregnant women on the female reproductive system and long acting contraceptives (LAC) including female sterilization by tubal ligation (locally known as “steri”) and intra-uterine device (IUD). Implants were not the focus of the discussion due to their high cost locally but this did not prevent discussion of them during FGDs. A simple picture of the female reproductive system (screened for acceptability by the facilitator, Supplementary file) and an IUD (unused, expired) were passed around the group and used to stimulate discussion. Participants were asked if they knew what the different parts of the female reproductive system were; if they knew what an IUD was and where an IUD would be placed, and which part of the reproductive system was operated on for sterilization. The dimensions of the picture were made to fit the IUD so women could try out different placements of the IUD onto the picture similar to a jigsaw puzzle.

A scenario that is repeatedly observed in practice was used to generate discussion on why high parity women at increased risk of maternal mortality fail to obtain FP [4]. This scenario involved a 35 year old highly parous woman (delivers her 6th alive child) who says she has finished her family, at the post-partum counseling with the midwife she says she wants sterilization, however at two months post-partum she says she cannot come back to the clinic.
