# Supplementary material for: Family planning knowledge, attitudes and practices in refugee and migrant pregnant and post-partum women on the Thailand-Myanmar border – a mixed methods study
Source: Reprod Health. 2016 Aug 19;13:94. doi: 10.1186/s12978-016-0212-2 (PMC4992227; doi:10.1186/s12978-016-0212-2)
Supplement: Additional file 3: Table S1. — Contraceptive uptake in Maela Camp 2013 to 2015. (PDF 214 kb) [file 12978_2016_212_MOESM3_ESM.pdf]

Supplementary Table. Contraceptive uptake in Maela Camp 2013 to 2015.

Type contraceptive	User type	2013	2014	2015
Injectable	New n, (% of total new users)	902, (35.2%)	676 (41.8%)	663 (39.9%)
	Continuing	2,305	1,664	1584
Pill	New n, (% of total new users)	1,118 (43.6%)	547 (33.9%)	513 (30.9%)
	Continuing	2,650	1,643	1064
Male-Condom	New n, (% of total new users)	338, (13.2%)	218, (1.3%)	123, (7.4%)
	Continuing	494	291	145
Female-Condom	New n, (% of total new users)	-	-	0
	Continuing	-	-	0
IUD[1]	New n, (% of total new users)	70, (2.7%)	86, (5.3%)	267, (16.1%)
	Continuing	11	1	0
Implant	New n, (% of total new users)	38 (1.5%)	27 (1.7%)	6 (0.4%)
	Continuing	19	-	0
Female Sterilization[1]	New n, (% of total new users)	95 (3.7%)	61 (3.8%)	90 (5.4%)
	Continuing	-	-	0
Vasectomy	New n, (% of total new users)	4 (1.6%)	-	0
	Continuing	-	-	0
Total	New	2,565	1,615	1662
FP clients	Continuing	5,479	3,599	2793

[1]PPAT and SMRU
